# Diaphragmatic function assessed during an SBT trial using m-mode ultrasonography and MIP: a pilot study

**DOI:** 10.1186/2197-425X-3-S1-A455

**Published:** 2015-10-01

**Authors:** S Spadaro, F Dalla Corte, V Alvisi, T Mauri, V Cricca, G Biondi, C Rizzuto, G Valpiani, F Barbieri, A Ferrararese, R Ragazzi, CA Volta

**Affiliations:** University of Ferrara / Intensive Care Unit, Morphology Surgery and Experimental Medicine, Ferrara, Italy; University of Foggia, Medicina Sperimentale e Rigenerativa, Foggia, Italy; Department of Anesthesia, Critical Care and Emergency, University of Milano. Fondazione IRCCS Ca' Granda Ospedale Maggiore Policlinico, Milano, Italy

## Introduction

Acute respiratory failure (ARF) is characterised by a discrepancy between load imposed on respiratory muscles and their capacity. The evaluation of inspiratory muscles performance is based mainly on Maximum Inspiratory Pressure (MIP) calculation. However, MIP is determined by the contraction of all the inspiratory muscles and doesn't give information about diaphragmatic function. Recently the Diaphragmatic Displacement (DD) measured by ultrasonography has been introduced in the clinical practice to evaluate diaphragmatic function.

## Objectives

We hypothesized that a correlation should exist between MIP and DD: the higher the DD, the higher the muscle performance evaluated as MIP.

## Methods

ICU patients ventilated for at least 24 hours were enrolled if they met all the criteria for a spontaneous breathing trial. Exclusion criteria: age < 18 years; pregnancy;; presence of flail chest or rib fractures; pneumothorax; primary neuromuscular disease; the use of muscle-paralyzing agents within 48 hours before the study. Thirty minutes after start of the SBT, right hemi-diaphragm ultrasound scans were performed in the semi-recumbent position: DD was evaluated using a 3.5-5 MHz convex probe was placed between the eighth and tenth intercostal space, between the anterior axillary and the mid-axillary lines. Simultaneously, spirometric measurements of tidal volume (V_T_) and respiratory rate (RR) were performed and, finally, MIP was measured according to the technique described by Marini.

## Results

**Figure 1:**
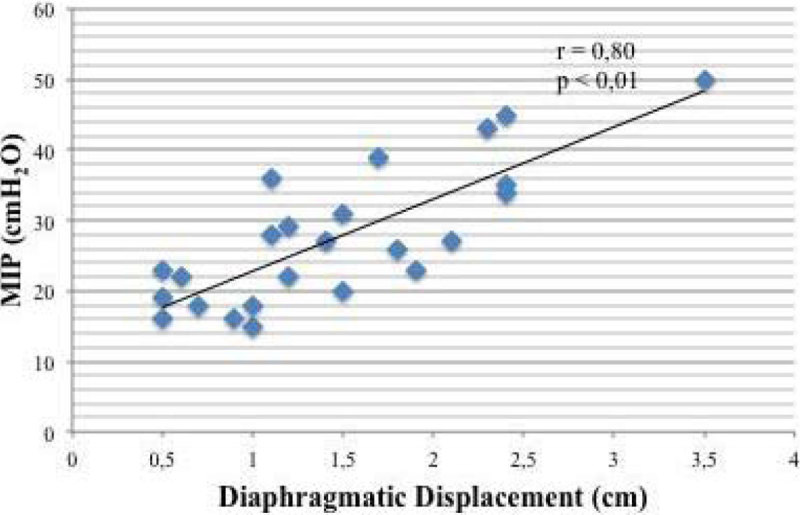
Figure 1

We included 24 patients (mean SAPSII: 36 ± 12) admitted in the ICU for sepsis (37%), post-operatory ARF (33%), COPD (17%) and heart failure (13%). There was a significant correlation between DD and MIP values (r = 0.80; p < 0.01) (Figure 1). Similarly, there was a significant correlation between DD and V_T_ (r = 0.63; p < 0.01) and RR (r = - 0.50; p < 0.05).

Different etiologies of ARF returned differences in baseline DD. Specifically, the Bonferroni post-hoc test demonstrated that patients with sepsis evidenced lower DD than the other patients (p < 0.01).

## Conclusions

We demonstrated that DD measured by ultrasonography is a specific, non-invasive and rapid diagnostic procedure that correlates well with muscle performance assessed by MIP determination, DD does not need patients' collaboration, as it is for MIP.

